# Outcome of an Extensive Mid‐Root Perforation With Unintentional Bioceramic Extrusion: A Case Report With 2‐Year Recall Using CBCT

**DOI:** 10.1155/crid/8877456

**Published:** 2026-06-10

**Authors:** Laila S. Almufleh

**Affiliations:** ^1^ Department of Conservative Dental Sciences, College of Dentistry, Prince Sattam Bin Abdulaziz University, Al-Kharj, Saudi Arabia, psau.edu.sa

**Keywords:** Bio-C repair, bioceramic materials, CBCT, iatrogenic perforation, mid-root perforation, root perforation repair

## Abstract

**Background:**

Iatrogenic root perforation is a serious complication that can adversely influence the prognosis of endodontic treatment. Premixed bioceramic repair materials have emerged as promising alternatives to mineral trioxide aggregate because of their favorable handling characteristics, biocompatibility, and bioactivity. This case report describes the 2‐year clinical and radiographic outcome of a large mid‐root perforation repaired nonsurgically with a premixed bioceramic material despite significant unintended extrusion into the periradicular tissues.

**Case Report:**

A 34‐year‐old man developed an iatrogenic mid‐root perforation in the middle third of the distal root of the mandibular left first molar during postspace preparation. The perforation was repaired immediately under magnification using a premixed tricalcium silicate‐based material (Bio‐C Repair). Postoperative imaging showed a well‐condensed repair with extrusion of the material beyond the root surface. CBCT assessment demonstrated a perforation diameter of 3.5 mm and material extrusion of 3 mm into the periradicular area. At 24 months, the tooth was functional and asymptomatic, with no tenderness to percussion or palpation and normal periodontal probing depths. Periapical radiography and CBCT demonstrated complete healing of the previous periapical lesion and no evidence of furcal pathosis.

**Conclusion:**

Within the limitations of a single case report, nonsurgical repair of a large mid‐root perforation with a premixed bioceramic material was associated with favorable clinical and radiographic healing, despite significant unintended material extrusion. This case supports the view that extrusion of bioceramic repair material does not necessarily preclude a favorable outcome.

## 1. Introduction

Root perforation is a communication between the root canal system and the external tooth surface that may occur pathologically or as an iatrogenic complication during endodontic treatment [1–4]. Its clinical significance lies in the potential for bacterial contamination, periodontal destruction, and compromised prognosis, particularly when diagnosis and repair are delayed [5]. Prognosis is influenced by several factors, including perforation size, location, time to repair, and the material used for sealing [3, 6].

Calcium silicate‐based materials have become central to perforation repair because of their biocompatibility, sealing ability, and bioactive properties [7, 8]. Premixed bioceramic materials may offer additional practical advantages in handling and delivery, but orthograde repair of large defects may be accompanied by material extrusion into the periradicular tissues [7, 9, 10].

Although previous reports have described favorable healing despite extrusion of calcium silicate‐based materials [11–13], additional case‐based evidence remains valuable, particularly when supported by long‐term clinical and radiographic follow‐up. This report describes the nonsurgical management of a large iatrogenic mid‐root perforation in a mandibular first molar repaired with a premixed bioceramic material, where CBCT demonstrated a perforation diameter of 3.5 mm and extrusion depth of 3 mm, with favorable healing observed at 24 months.

## 2. Case Report

A 34‐year‐old male with a noncontributory medical history (American Society of Anesthesiologists Physical Status I) presented to the undergraduate clinic at the College of Dentistry, Prince Sattam Bin Abdulaziz University for endodontic treatment of the left mandibular first molar (Tooth #36, FDI notation). Based on clinical and radiographic findings, a preoperative diagnosis of pulp necrosis with symptomatic apical periodontitis was established (Figure 1).

**Figure 1:**
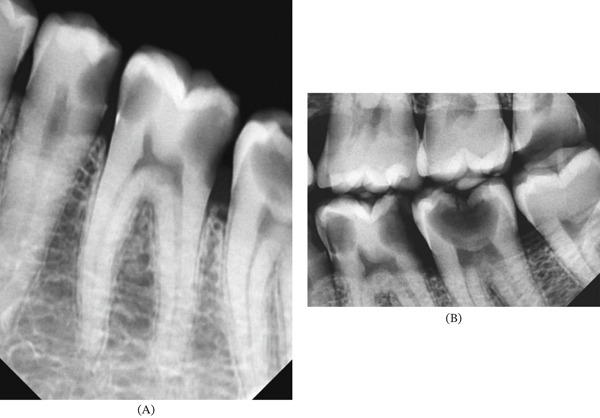
Preoperative radiographs. (A) Periapical radiograph and (B) left molar bitewing radiograph.

Nonsurgical root canal treatment was completed in a single visit. After administration of local anesthesia (2% lidocaine with 1:100,000 epinephrine; Dentsply Sirona, United States) and rubber dam isolation, an access cavity was prepared using a high‐speed No. Two diamond round bur (Dentsply Sirona, United States). Working length was determined using an electronic apex locator (E‐Pex, Eighteeth, China) and confirmed radiographically (Figure 2). Chemomechanical preparation was performed using a crown‐down technique with ProTaper Gold rotary files (Dentsply Sirona, United States) up to size 25/08 in the mesial canals and 30/09 in the distal canal, with copious irrigation using 5.25% sodium hypochlorite throughout instrumentation. After final irrigation, the canals were dried with sterile paper points (Dentsply Sirona, United States) and obturated with gutta‐percha (Dentsply Sirona, United States) and a bioceramic sealer (Bio C Sealer; Angelus, Brazil) using the single‐cone technique (Figure 2). A temporary glass ionomer restoration (3 M, United States) was placed with a polytetrafluoroethylene tape spacer to facilitate future access.

**Figure 2:**
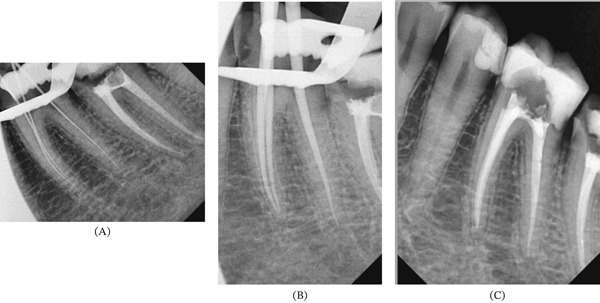
Treatment radiographs. (A) Working length radiograph, (B) master cone radiograph, and (C) postobturation radiograph.

At the subsequent visit, 1 week later, an iatrogenic perforation occurred in the middle third of the distal root during postspace preparation with a Size 4 Gates Glidden drill (Dentsply Sirona, United States) (Figure 3). The perforation was immediately managed by the supervising endodontist during the same visit. Repair was performed under rubber dam isolation and a dental operating microscope (Zumax, China). The site was irrigated with sterile normal saline and dried with size 70 paper points. A premixed tricalcium silicate‐based cement (Bio‐C Repair; Angelus, Brazil) was delivered to the defect using a MAP One carrier (Dentsply Maillefer, Switzerland) and condensed with a Schilder plugger (Dentsply Caulk, Milford, United States). A postrepair periapical radiograph showed a well‐condensed repair mass, although significant unintended extrusion of the bioceramic material into the periradicular space was observed (Figure 4). Subsequent CBCT assessment demonstrated that the perforation had a maximum diameter of approximately 3.5 mm and that the extruded material extended approximately 3 mm beyond the external root surface. The patient was informed of the procedural complication and the associated guarded long‐term prognosis and was reassured regarding the planned management and follow‐up.

**Figure 3:**
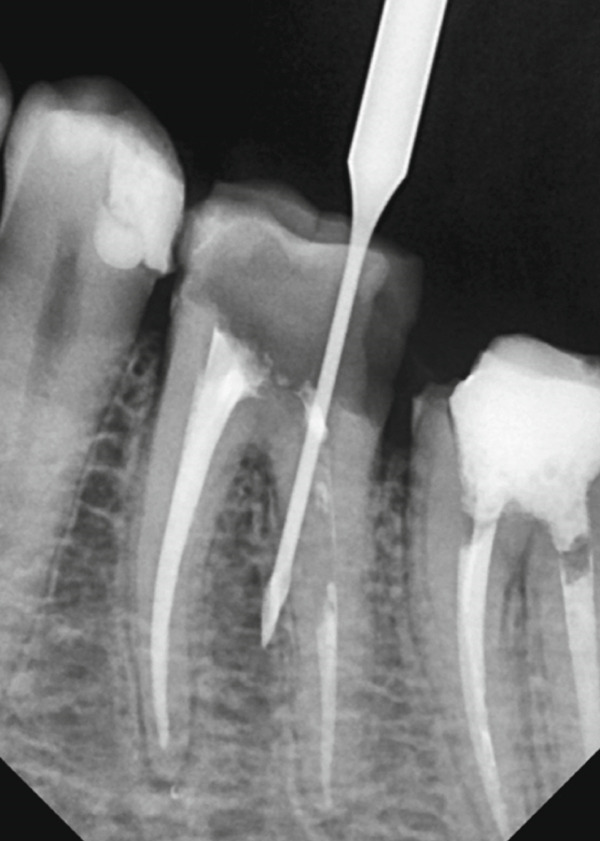
Periapical radiograph showing the perforation of the mesial wall of the distal root in the middle third with a Gates Glidden drill Size 4.

**Figure 4:**
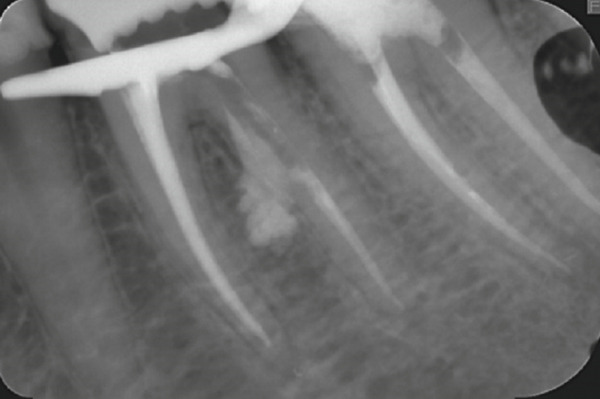
Periapical radiograph after perforation repair showing a well‐condensed mass of repair material at the perforation site, as well as significant extrusion of the bioceramic material into the periradicular area.

Following perforation repair, a flexible glass fiber post (3 M, United States) was fitted under rubber dam isolation and luted with a self‐adhesive dual‐cured resin cement (RelyX Unicem, 3 M, United States). Core buildup was then completed using the same material. A postcementation radiograph was taken to verify the integrity of the restoration (Figure 5). One week later, the tooth was prepared for a zirconium‐oxide ceramic crown (Cercon; Dentsply Sirona, United States). Final impressions were made with polyvinyl siloxane material (Ultradent, United States) using a two‐stage double‐mix technique. A provisional crown was fabricated and temporarily cemented, after which the definitive zirconium‐oxide crown was polished and cemented using self‐adhesive dual‐cure resin cement (RelyX Unicem, 3 M, United States).

**Figure 5:**
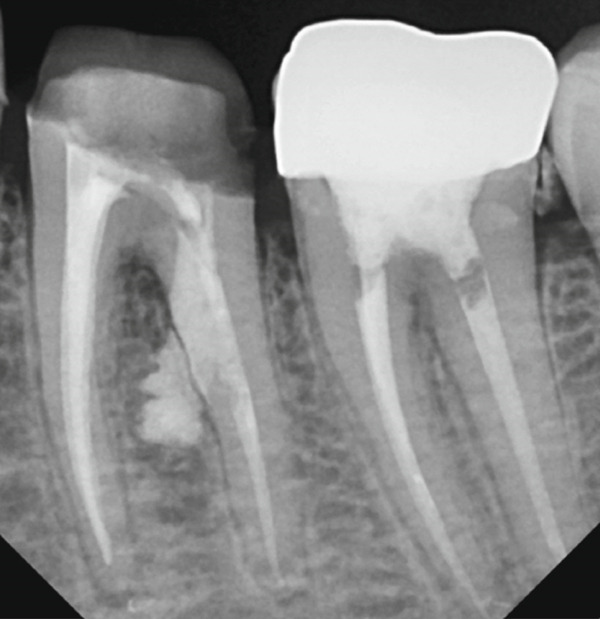
Periapical radiograph after fiberpost placement and core build up.

All periapical radiographs were obtained using a beam‐aiming device with a Soredex Minray intraoral X‐ray unit and a Carestream RVG 5200 digital sensor, operated at 66 kV, 7.5 mA, and 0.10‐s exposure time using the paralleling technique. Images were stored as uncompressed files.

At the 24‐month follow‐up, the patient remained asymptomatic. Clinical examination of Tooth #36 showed no signs of inflammation, no tenderness to percussion or palpation, and periodontal probing depths within normal limits. Radiographic assessment included periapical radiography and CBCT. Follow‐up CBCT scan was acquired using a Carestream 2100 unit with a 4 × 4 cm field of view, 0.1‐mm voxel size, 90 kVp, 6.5 mA, and a 15‐s exposure. Images were reconstructed at 0.125‐mm slice intervals with 1.5‐mm slice thickness using CS 3D Imaging Software (Carestream). Periapical radiography and CBCT demonstrated complete resolution of the previous periapical radiolucency and absence of furcal radiolucency, indicating successful osseous healing despite the initial extrusion of the repair material (Figures 6 and 7).

**Figure 6:**
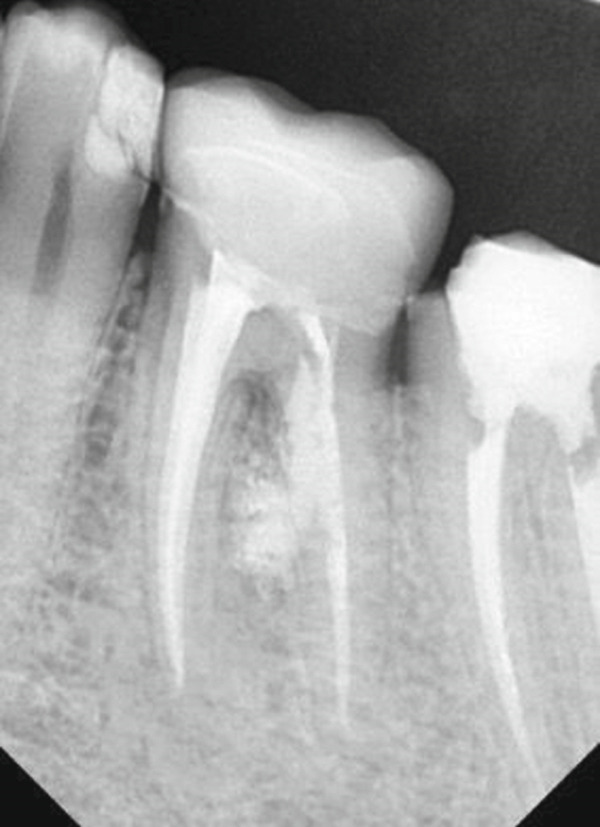
Follow‐up periapical radiograph at 24 months showing signs of healing of periapical bony lesions and absence of bony changes associated with the perforation area.

**Figure 7:**
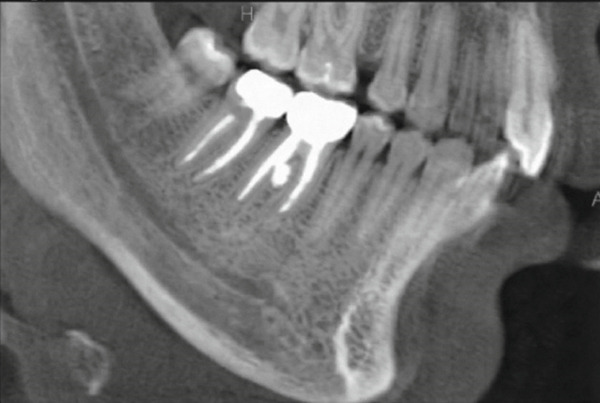
Follow‐up CBCT at 24 months confirming healing of apical radiolucency and absence of any associated bony changes in the perforation site.

Written informed consent was obtained from the patient for publication of this case report and the accompanying radiographic images. In accordance with institutional policy, formal ethical approval was not required for this single‐patient case report.

## 3. Discussion

Root perforation remains one of the most challenging complications in endodontic treatment because its prognosis is influenced by multiple interacting factors, including the size and location of the defect, the time elapsed before repair, and the material used for sealing [14–17]. In the present case, favorable healing was observed despite the presence of a large mid‐root perforation and unintentional extrusion of bioceramic material into the surrounding tissues. This outcome may be attributed to early recognition of the perforation, immediate sealing of the defect, use of a bioactive repair material, and appropriate follow‐up.

The present case should be interpreted in the context of previous reports describing favorable healing after extrusion of calcium silicate‐based materials. Accordingly, its contribution lies not in absolute novelty, but in adding further clinical and radiographic evidence that such extrusion does not necessarily preclude periradicular healing when the case is appropriately managed and followed over time. In this regard, the report has practical relevance because it illustrates the clinical course, conservative follow‐up strategy, and favorable long‐term outcome.

An additional consideration in this case relates to procedural planning during postspace preparation. Postspace preparation in molar roots may be challenging because root concavities, canal angulation, and reduced dentin thickness can increase the risk of lateral deviation and perforation [4]. In the present case, the use of a Gates‐Glidden drill rather than a dedicated postsystem preparation instrument may also have influenced this risk, since most post systems are designed to be used with matched drills corresponding to the intended post geometry [18, 19]. These factors highlight the importance of appropriate case selection, instrument choice, and attention to root anatomy, particularly in posterior teeth [4]. Importantly, the present case also provides useful follow‐up information after immediate repair of an iatrogenic perforation. Although the favorable healing outcome should be interpreted within the limitations of a single case report, it adds clinically relevant information regarding the management and follow‐up of this type of complication.

Bioceramic calcium silicate‐based materials have become increasingly important in perforation management because of their biocompatibility, sealing ability, dimensional stability, and bioactivity [20, 21]. These materials promote the release of calcium ions and the formation of hydroxyapatite, which may support hard tissue deposition and periodontal healing [22]. Compared with conventional MTA, premixed bioceramics may also offer improved handling and easier delivery, particularly in challenging clinical situations [23]. Such properties may help explain the favorable response observed in the present case, even in the presence of material extrusion.

In the present case, Bio‐C Repair, a premixed calcium silicate‐based bioceramic material, was used for perforation repair. Its syringe‐based delivery system facilitates handling and placement, whereas available evidence suggests biocompatibility and bioactivity comparable with those of white MTA and other established calcium silicate‐based materials [24–27]. However, published evidence specifically addressing its use in perforation management remains limited [28].

Nevertheless, extrusion of repair material beyond the perforation site should not be regarded as a desirable outcome. Excess material in the periodontal tissues may complicate healing, interfere with tissue adaptation, and potentially compromise prognosis, depending on the extent and location of extrusion and the host tissue response [29–33]. In the current case, however, the absence of symptoms, resolution of the radiolucency, and re‐establishment of normal periodontal structures over time suggested that the extruded material was well tolerated.

The use of magnification and illumination likely contributed significantly to treatment success. Management of mid‐root perforations requires precise visualization to identify the defect, control bleeding, and place the repair material accurately [34]. The dental operating microscope improves visualization of the operative field and enhances the clinician′s ability to manage procedural complications conservatively [34–36]. In the present case, magnification facilitated accurate localization and repair of the perforation, which may have reduced additional procedural trauma and improved sealing quality.

Coronal restoration is another critical determinant of long‐term outcome. A well‐sealed and structurally protective final restoration limits coronal microleakage and supports the mechanical survival of endodontically treated teeth [37, 38]. The provision of cuspal coverage in this case may have contributed to the favorable long‐term result by protecting the remaining tooth structure and improving fracture resistance. This is consistent with previous evidence showing improved survival of root‐filled posterior teeth restored with cuspal coverage [38–41].

Cone beam computed tomography has emerged as a valuable diagnostic tool for the objective assessment of endodontic treatment outcomes because it provides a three‐dimensional view of the tooth and surrounding structures [42–46]. Conventional periapical radiography has recognized limitations in detecting periapical lesions, particularly when lesions are small, confined to cancellous bone, or obscured by overlying cortical structures [45, 47–50]. In the present case, CBCT enabled more precise evaluation of healing by confirming re‐establishment of the periodontal ligament space and integrity of the cortical plate. This added confidence to the interpretation that the extruded material had not prevented tissue healing.

Overall, this case suggests that favorable healing may be achieved after repair of a large iatrogenic mid‐root perforation, even in the presence of extruded calcium silicate‐based material. The favorable outcome highlights the importance of timely intervention, appropriate material selection, adequate magnification, and careful follow‐up. Further well‐designed clinical studies are needed to better define prognostic factors and compare outcomes among different calcium silicate‐based repair materials.

## 4. Conclusion

At the 24‐month follow‐up, favorable healing was observed after nonsurgical repair of a large iatrogenic mid‐root perforation, despite unintended extrusion of premixed bioceramic material. This case also emphasizes the importance of careful treatment planning, appropriate instrument selection, and consideration of root anatomy during postspace preparation. When perforation occurs, early recognition and prompt management remain essential. Within the limitations of a single case report, conservative repair with a premixed bioceramic material may be associated with favorable healing in selected cases, although further clinical evidence is required.

## Funding

No funding was received for this manuscript.

## Conflicts of Interest

The author declares no conflicts of interest.

## Data Availability

The data that support the findings of this study are available on request from the corresponding author. The data are not publicly available due to privacy or ethical restrictions.
